# FluA-p score: a novel prediction rule for mortality in influenza A-related pneumonia patients

**DOI:** 10.1186/s12931-020-01379-z

**Published:** 2020-05-08

**Authors:** Liang Chen, Xiudi Han, Yan Li Li, Chunxiao Zhang, Xiqian Xing

**Affiliations:** 1 Department of Infectious Diseases, Beijing Jishuitan Hospital, 4th Medical College of Peking University, Beijing, China; 2 Department of Pulmonary and Critical Care Medicine, Qingdao Municipal Hospital, Qingdao City, Shandong Province China; 3 Department of Infectious Diseases and Clinical Microbiology, Beijing Chao-Yang Hospital, Capital Medical University, Beijing, China; 4 Department of Pulmonary and Critical Care Medicine, Beijing Huimin Hospital, Beijing, China; 5 Department of Pulmonary and Critical Care Medicine, the 2nd People’s Hospital of Yunnan Province, Kunming City, Yunnan Province China

**Keywords:** Influenza, Pneumonia, Mortality, Prediction rule

## Abstract

**Background:**

The pneumonia severity index (PSI) and the CURB-65 (confusion, urea, respiratory rate, blood pressure, age ≥ 65 years) score have been shown to predict mortality in community-acquired pneumonia. Their ability to predict influenza-related pneumonia, however, is less well-established.

**Methods:**

A total of 693 laboratory-confirmed FluA-p patients diagnosed between Jan 2013 and Dec 2018 and recruited from five teaching hospitals in China were included in the study. The sample included 494 patients in the derivation cohort and 199 patients in the validation cohort. The prediction rule was established based on independent risk factors for 30-day mortality in FluA-p patients from the derivation cohort.

**Results:**

The 30-day mortality of FluA-p patients was 19.6% (136/693). The FluA-p score was based on a multivariate logistic regression model designed to predict mortality. Results indicated the following significant predictors (regression statistics and point contributions toward total score in parentheses): blood urea nitrogen > 7 mmol/L (*OR* 1.604, *95% CI* 1.150–4.492, *p* = 0.040; 1 points), pO_2_/F_i_O_2_ ≤ 250 mmHg (*OR* 2.649, *95% CI* 1.103–5.142, *p* = 0.022; 2 points), cardiovascular disease (*OR* 3.967, *95% CI* 1.269–7.322, *p* < 0.001; 3 points), arterial PH < 7.35 (*OR* 3.959, *95% CI* 1.393–7.332, *p <* 0.001; 3 points), smoking history (*OR* 5.176, *95% CI* 2.604–11.838, *p* = 0.001; 4 points), lymphocytes < 0.8 × 10^9^/L (*OR* 8.391, *95% CI* 3.271–16.212, *p* < 0.001; 5 points), and early neurominidase inhibitor therapy (*OR* 0.567, *95% CI* 0.202–0.833, *p* = 0.005; − 2 points). Seven points was used as the cut-off value for mortality risk stratification. The model showed a sensitivity of 0.941, a specificity of 0.762, and overall better predictive performance than the PSI risk class (AUROC = 0.908 vs 0.560, *p* < 0.001) and the CURB-65 score (AUROC = 0.908 vs 0.777, *p* < 0.001).

**Conclusions:**

Our results showed that a FluA-p score was easy to derive and that it served as a reliable prediction rule for 30-day mortality in FluA-p patients. The score could also effectively stratify FluA-p patients into relevant risk categories and thereby help treatment providers to make more rational clinical decisions.

## Background

Influenza is a common contagious respiratory disease and influenza-related epidemics and pandemics have occurred all over the world [1, 2]. Despite advances in medical technology and greater economic development in many countries, influenza still causes numerous hospitalizations and is associated with considerable mortality [3–5]. Each year, 10–20% of the global population experiences symptomatic influenza, including 3–5 million cases of severe illness and 290–650 thousand deaths [6]. For these reasons, influenza is regarded as the greatest threat to global health in the twenty-first century [7].

Patients infected with influenza may exhibit a broad spectrum of clinical symptoms, ranging from self-limited upper respiratory tract illness to severe pneumonia [8, 9]. Influenza-related pneumonia (Flu-p), including primary viral pneumonia and secondary bacterial pneumonia, is the major cause of influenza-associated hospitalizations and deaths [10]. Primary influenza pneumonia and post-influenza secondary bacterial pneumonia are distinct pathologies but difficult to distinguish clinically. The pathogenesis of primary influenza pneumonia shows diffuse alveolar damage associated with haemorrhage and necrotising bronchiolitis, and the secondary bacterial pneumonia presents with neutrophil influx, loss of alveolar architecture and consolidation [10]. When the diagnosis of pneumonia is confirmed, the first priority is to assess the degree of disease severity. Several prediction rules have been established to help clinicians predict the mortality rate of patients with pneumonia. Scores on the CURB-65 (confusion, urea, respiratory rate, blood pressure, age ≥ 65 years) and the pneumonia severity index (PSI) are the most widely used indices to predict 30-day mortality rates for patients diagnosed with community-acquired pneumonia [11, 12]. However, the validity of these two measures for use with Flu-p patients is questionable [13, 14]. Some variables that might be more useful in predicting severe influenza include PO_2_/FiO_2_ and lymphocyte counts [15, 16]. But to our knowledge, standard decision rules using these (and perhaps other) variables to predict the extent of Flu-p severity have yet to be developed.

In an effort to remedy this situation we conducted a multicenter, retrospective study with the principal aim being to develop an easy-to-use and accurate severity assessment tool to predict the 30-day mortality rate of patients with influenza A-related pneumonia (FluA-p). Our assessment tool is designed to have greater predictive power than either CURB-65 or PSI scores.

## Methods

### Study design and patient recruitment

Hospitalised patients who tested positive for influenza A virus RNA at the Microbiology Labs of five tertiary hospitals in China from 1st Jan 2013 to 31st Dec 2018 were screened for inclusion (the information for the participating centers is contained in Supplementary material 1). Patients with laboratory-confirmed Flu-p were included. Exclusion criteria for the patients were as follows [17]: (i) Younger than 14 years old; (ii) pneumonia whose onset was not in the community (i.e., pneumonia onset ≥48 h after admission and hospitalised within the last 28 days); and (iii) immunocompromised status.

### Disease and treatment definitions

Patients with influenza-related pneumonia experienced disease onset during the influenza season and manifested with respiratory symptoms along with newly developed pulmonary infiltrates on chest radiographs. In addition, patients with influenza-related pneumonia tested positive for influenza virus RNA by reverse-transcription polymerase chain reaction (RT-PCR). The biological samples subjected to RT-PCR were respiratory specimens (i.e., nasal/nasopharyngeal swabs, sputum, bronchial aspirates or branchoalveolar lavage fluid). Community-acquired respiratory co-infections resulting from coinfected pathogens were identified using standard microbiologic procedures within the first 48 h after admission [18]. Early neuraminidase inhibitor (NAI) treatment was defined as any NAI (oseltamivir, zanamivir or peramivir) administered within 48 h after illness onset [19]. Systemic corticosteroid use was defined as at least one dose of any systemic corticosteroid administered during hospitalisation.

### Data collection

Data were retrospectively collected and included demographic information, chronic medical conditions (Supplementary material 2), baseline clinical characteristics (clinical symptoms, vital signs, laboratory and radiological findings), illness severity of pneumonia on admission (CURB-65 and PSI scores), community-acquired respiratory coinfections (Supplementary material 3), clinical management (administration of NAI, systemic corticosteroid use, invasive and non-invasive mechanical ventilation, admittance to the intensive care unit (ICU)), and 30-day mortality rate.

### Statistical analysis

All of 693 FluA-p patients were divided into a derivation cohort (494 patients from 2013 to 2016) and a validation cohort (199 patients from 2017 to 2018). The derivation cohort was used to establish the statistical model, and the validation cohort was used to validate the model.

According to the survival status at 30 days post-admission, the 693 patients were divided into surviving and deceased groups. Baseline characteristics of these two groups were compared. Variables with *p*-values < 0.1 in the univariate analyses were entered into a backward stepwise logistic regression model to explore risk factors for 30-day mortality. For pragmatic reasons, the score for each predictor was assigned an integer value relative to the regression coefficient (β). A cut-off point was designated following Youden’s index from the receiver operating characteristic (ROC) curve. A Kaplan-Meier analysis was performed to compare the difference in 30-day mortality rates between the low-risk and high-risk groups according to the designated cut-off value. Performance of the cut-off score was assessed by measuring the area under the ROC curve (AUROC) and by calculating measures of sensitivity and specificity.

The data were analysed for normality using a Kolmogorov–Smirnov test. In presenting our results, variables with a normal distribution are shown as the mean ± standard deviation. Those variables with a non-normal distribution are expressed as medians. Categorical variables were analysed using either the Chi-square test or Fisher’s exact test. Continuous variables were analysed using either Student’s *t* test or the Mann–Whitney *U* test. For all analyses, a two-tailed *P*-value < 0.05 was considered statistically significant. All statistical analyses were performed using either SPSS version 22.0 or MedCalc version 19.0.

## Results

### Screening process

**Fig. 1:**
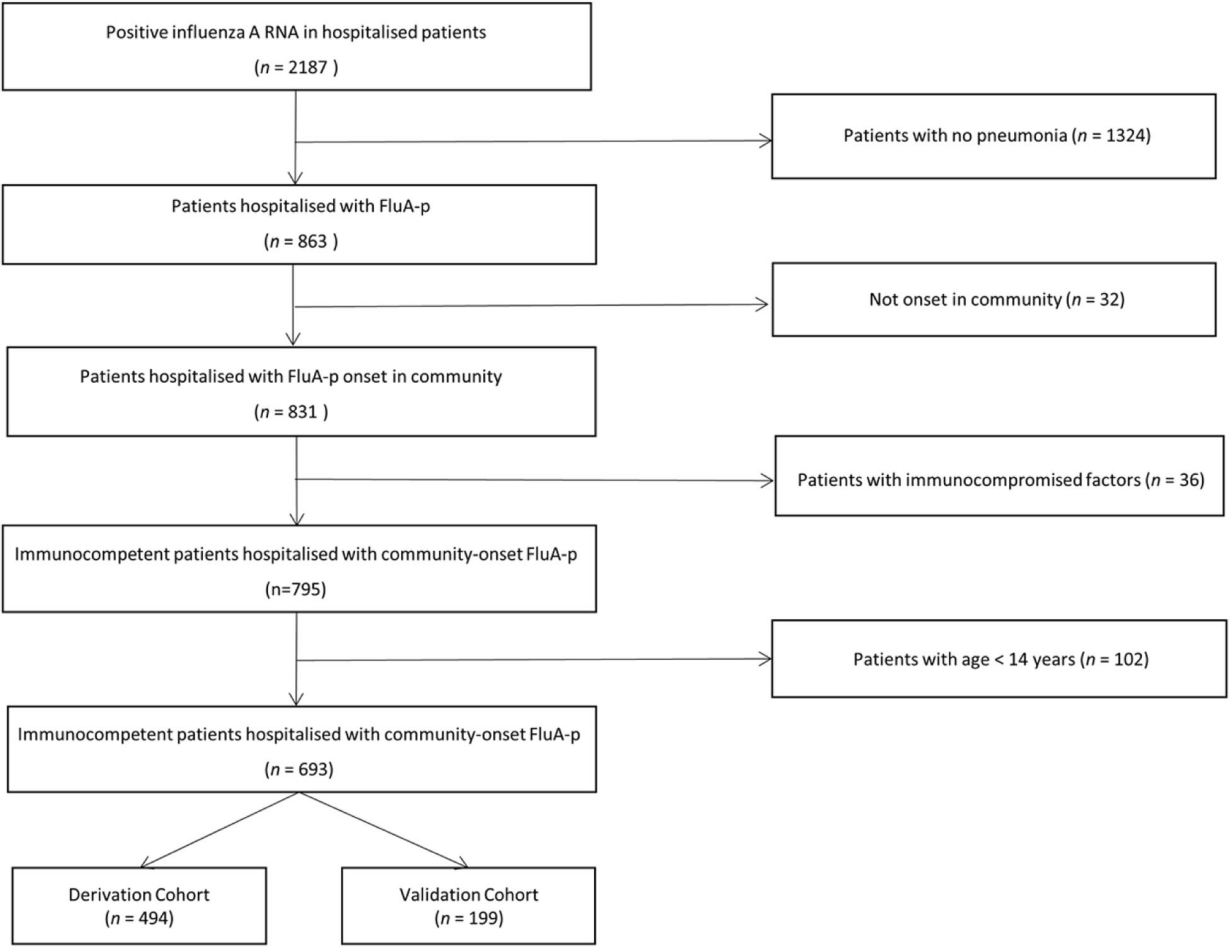
Patient screening algorithm for FluA-p

We screened 2187 hospitalised patients who tested positive for influenza A RNA. Overall, 693 immunocompetent adult and adolescent patients hospitalised with FluA-p were included in the final analysis (Fig. 1). Among these patients, 38.1% (264/693) were infected with A(H1N1)pdm09 and 11.0% (76/693) were infected with A(H3N2). In addition, 50.9% (353/693) of patients were infected with an unclassified influenza subtype. Because not all of the five hospitals in our study carried out influenza subtype tests, to identify the subtypes of influenza A using RT-PCR, subtype-specific primers were developed.

### Overview of FluA-p patients

Overall, 39.2% (272/693) of patients were above 65 years old and 66.5% (461/693) of patients were male. 35.1% (243/693) of patients had a history of smoking. Cardiovascular disease (19.6%), diabetes mellitus (13.3%) and cerebrovascular disease (10.4%) were the most common chronic medical conditions. Respiratory rates ≥30 beats/min and mental confusion could be seen in 17.5% (121/693) and 4.6% (32/693) of patients, respectively. Only 1.2% (8/693) of patients had SBP < 90 mmHg. 26.9% (172/639) of patients had pO_2_/FiO_2_ ≤ 250 mmHg (Table 1).

**Table 1:** Comparison of clinical features between deceased and survival patients

Variable	Total (*n* = 693)	Deceased group (*n* = 136)	Survival group (*n* = 557)	*p*-value
Male (*n*, %)	461 (66.5)	92 (67.6)	369 (66.2)	0.757
Age ≥ 65 years (*n*, %)	272 (39.2)	60 (44.1)	212 (38.1)	0.195
Obesity (*n*, %)^a^	48 (6.9)	0 (0.0)	48 (8.6)	**< 0.001**
Pregnancy (*n*, %)	8 (1.2)	0 (0.0)	8 (1.4)	0.338
Smoking history (*n*, %)^a^	243 (35.1)	68 (50.0)	175 (31.4)	**< 0.001**
Comorbidities (*n*, %)				
Cardiovascular disease^a^	136 (19.6)	48 (35.3)	88 (15.8)	**< 0.001**
Diabetes mellitus	92 (13.3)	14 (10.3)	78 (14.0)	0.253
Cerebrovascular disease	72 (10.4)	10 (7.4)	62 (11.1)	0.195
COPD^a^	40 (5.8)	3 (2.2)	37 (6.6)	**0.047**
Asthma	19 (2.7)	2 (1.5)	17 (3.1)	0.222
Chronic kidney disease	16 (2.3)	6 (4.4)	10 (1.8)	0.139
Malignant solid tumor	16 (2.3)	0 (0.0)	16 (2.9)	0.193
Clinical and radiologic characteristics (*n*, %)				
Respiratory rates ≥30 times/min	121 (17.5)	25 (18.4)	96 (17.2)	0.752
Mental confusion^a^	32 (4.6)	32 (23.5)	0 (0.0)	**< 0.001**
SBP < 90 mmHg	8 (1.2)	0 (0.0)	8 (1.4)	0.338
Leukocytes > 10 × 10^9^/L^a^	118 (17.0)	42 (30.9)	76 (13.6)	**< 0.001**
Lymphocytes < 0.8 × 10^9^/L^a^	299/677 (44.2)	120 (88.2)	179/541 (33.1)	**< 0.001**
Hb < 100 g/L^a^	69 (10.0)	34 (25.0)	35 (6.3)	**< 0.001**
ALB < 35 g/L^a^	58/639 (9.1)	12/131 (9.2)	46/508 (9.1)	0.970
BUN > 7 mmol/L^a^	183/685 (26.7)	97 (71.3)	86/549 (15.7)	**< 0.001**
BG > 14 mmol/L	8 (1.2)	0 (0.0)	8 (1.4)	0.288
Arterial PH < 7.35^a^	120/639 (18.8)	60 (44.1)	60/503 (11.9)	**< 0.001**
pO_2_/F_i_O_2_ ≤ 250 mmHg^a^	172/639 (26.9)	28 (20.6)	144/503 (28.6)	0.061
Multilobar infiltrates^a^	546 (78.8)	120 (88.2)	426 (76.5)	**0.003**
Pleural effusion^a^	120 (17.3)	36 (26.5)	84 (15.1)	**< 0.001**
Coinfections (*n*, %)^a^	265 (38.2)	84 (61.8)	181 (32.5)	**< 0.001**
Early NAI use (*n*, %)^a^	232 (33.5)	60 (43.4)	172 (30.9)	**0.003**
Systemic corticosteroid use (*n*, %)^a^	132 (19.0)	60 (44.1)	72 (12.9)	**< 0.001**
Noninvasive ventilation (*n*,%)	159 (22.9)	71 (52.2)	88 (15.8)	**< 0.001**
Invasive ventilation (*n*,%)	158 (22.8)	86 (63.2)	72 (12.9)	**< 0.001**
Admittance to ICU (*n*,%)	176 (25.4)	92 (67.6)	84 (15.1)	**< 0.001**

The bolded values are *p*-values < 0.05, which represented significant differences between survival group and deceased group

*COPD* chronic obstructive pulmonary disease, *SBP* systolic blood pressure, *Hb* hemoglobin, *ALB* albumin, *BUN* blood urea nitrogen, *BG* blood glucose, *pO*_*2*_*/FiO*_*2*_ arterial pressure of oxygen/fraction of inspiration oxygen, *NAI* neuraminidase inhibitor

^a^variables cited in the table above were the candidates which were entered into the multivariate logistic regression model

Almost 40 % (38.2%, 265/693) of patients were coinfected with other community-acquired pathogens. *Streptococcus pneumoniae* (33.2%) was the most common coinfection, followed by *Klebsiella pneumoniae* (30.6%) and *Staphylococcus aureus* (20.4%) (Supplementary material 4).

All patients received antibiotic treatment within 48 h after admission (Supplementary material 5), and NAI therapy during the course of the disease. Early NAI therapy and systemic corticosteroid use were administered in 33.5% (232/693) and 19.0% (132/693) of patients, respectively. 22.8% (158/693) of patients received invasive ventilation, 25.4% (176/693) of patients were admitted to the ICU, and the 30-day mortality rate was 19.6% (136/693) (Table 1).

There were no significant differences in the demographic characteristics, clinical features, approach to clinical management, and treatment outcomes between patients in the derivation and validation cohorts (Supplementary material 6).

### Predicted and actual mortality in FluA-p patients stratified by CURB-65 score and PSI risk class

Supplemental material 7 shows the actual and predicted mortality rates stratified by PSI risk class and CURB-65 scores. For the 136 deceased patients, the proportions of patients with PSI risk I ~ V were 38.2% (52/136), 8.8% (12/136), 5.9% (8/136), 47.1% (64/136) and 0% (0/136), respectively; the proportions of patients with CURB-65 scores 0–5 were 0% (0/136), 66.9% (91/136), 12.5% (17/136), 0% (0/136) and 0% (0/136), respectively.

### Risk factors for 30-day mortality

Following the procedures described in the Statistical Analysis section, the following variables were entered into a backward stepwise logistic regression analysis: obesity, smoking history, cardiovascular disease, chronic pulmonary disease (COPD), altered mental status, leukocytes > 10 × 10^9^/L, lymphocytes < 0.8 × 10^9^/L, hemoglobin (Hb) < 100 g/L, albumin (ALB) < 35 g/L, blood urea nitrogen (BUN) > 7 mmol/L, arterial PH < 7.35, pO_2_/FiO_2_ ≤ 250 mmHg, multilobar infiltrates, pleural effusion, early NAI therapy, systemic corticosteroid use, and coinfections.

**Fig. 2:**
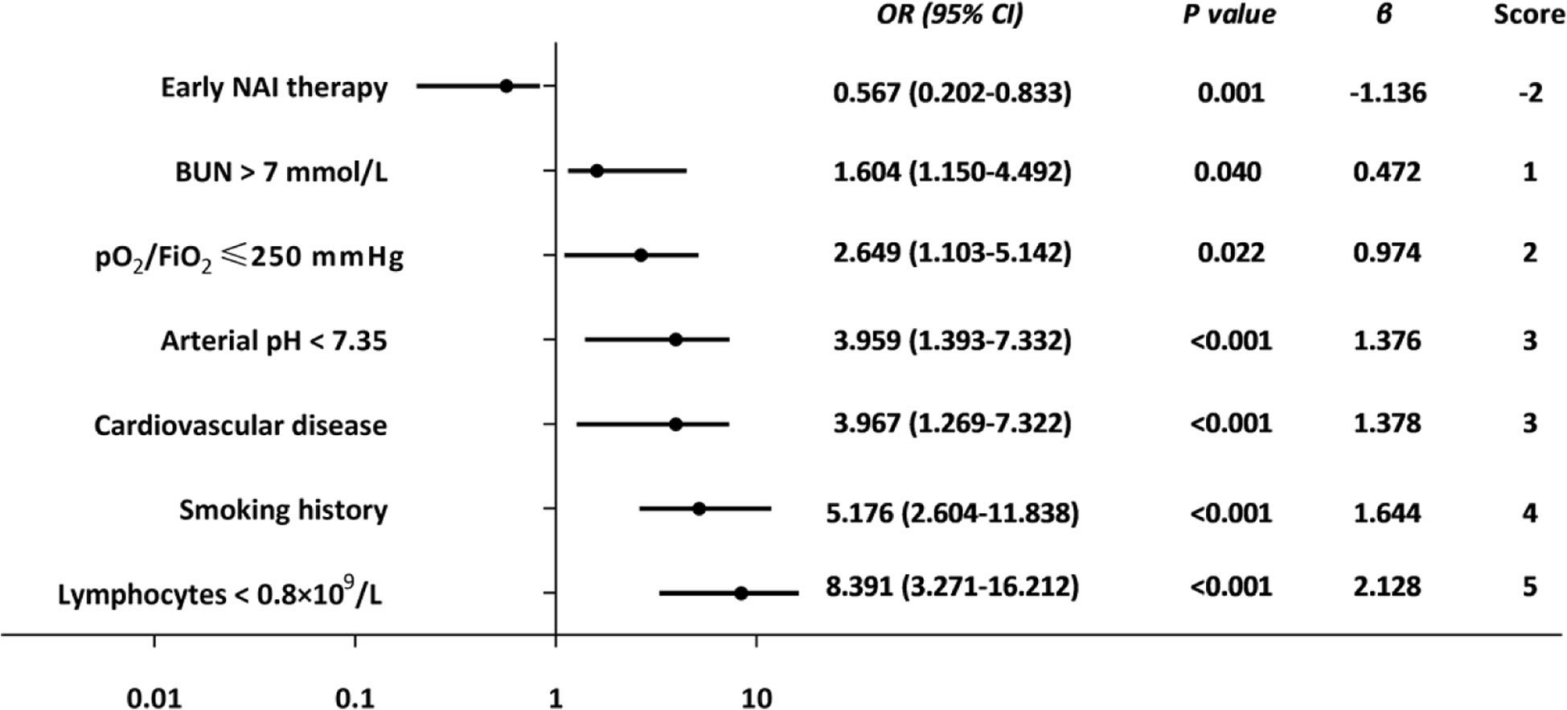
Multivariate analysis associated with mortality of FluA-p patients

A multivariate logistic regression model indicated that the following variables were significantly associated with 30-day mortality (see Fig. 2): BUN > 7 mmol/L (*OR* 1.604, *95% CI* 1.150–4.492, *p* = 0.040), pO_2_/F_i_O_2_ ≤ 250 mmHg (*OR* 2.649, *95% CI* 1.103–5.142, *p* = 0.022), cardiovascular disease (*OR* 3.967, *95% CI* 1.269–7.322, *p* < 0.001), arterial PH < 7.35 (*OR* 3.959, *95% CI* 1.393–7.332, *p* < 0.001), smoking history (*OR* 5.176, *95% CI* 2.604–11.838, *p* < 0.001), lymphocytes < 0.8 × 10^9^/L (*OR* 8.391, *95% CI* 3.271–16.212, *p* < 0.001) and early NAI therapy (*OR* 0.567, *95% CI* 0.202–0.833, *p* = 0.001).

### Comparison of severity scores for mortality prediction

**Fig. 3:**
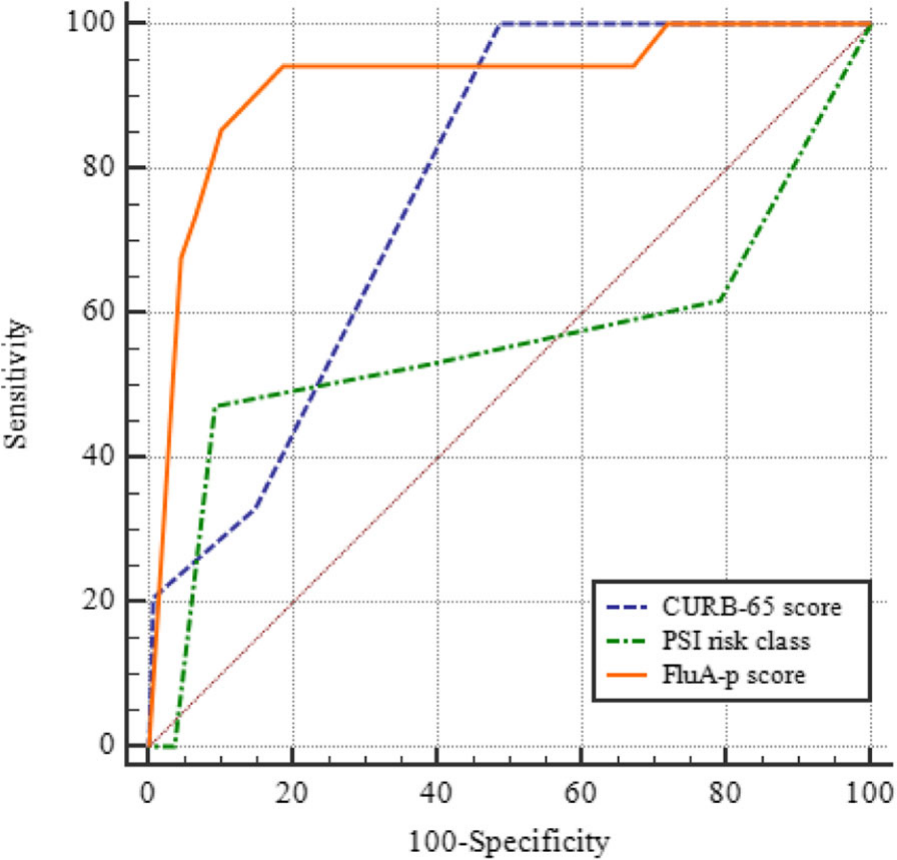
ROCs for mortality prediction of three severity scores in FluA-p patients

**Fig. 4:**
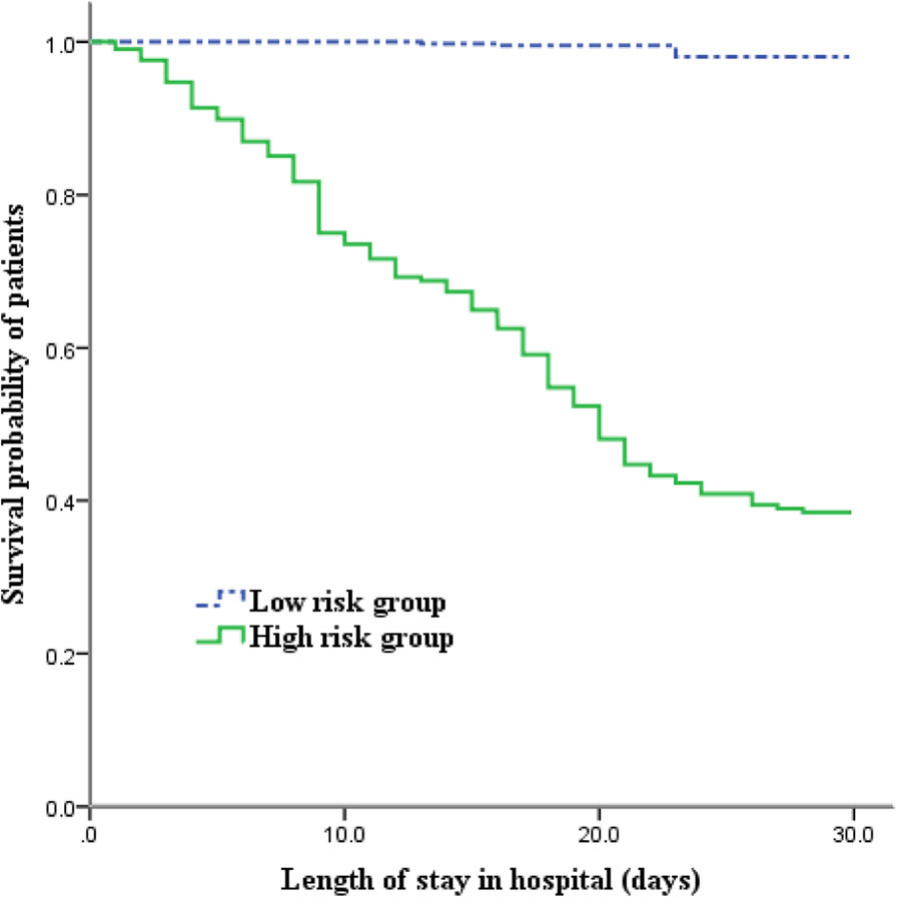
Survival of FluA-p patients by different levels of FluA-p scores. For 30-day mortality: FluA-p score < 7: Low risk; FluA-p score ≥ 7: High risk

In order to develop a simple and useful clinical predicting tool, relative weights were assigned according to the regression coefficient (*β*) of each categorical variable. Supplementary material 8 shows that the AUROC of the derivation cohort was 0.934 (*95% CI* 0.906–0.957), which was higher than the CURB-65 score (AUC = 0.813, *95% CI* 0.772–0.850, *p* < 0.001) and the PSI risk class (AUC = 0.577, *95% CI* 0.527–0.625, *p* < 0.001) (Supplemental Figure 1). Supplementary material 9 shows that the AUROC of the validation cohort was 0.846 (*95% CI* 0.781–0.897), which was higher than the CURB-65 score (AUC = 0.681, *95% CI* 0.604–0.752, *p* < 0.001) and the PSI risk class (AUC = 0.525, *95% CI* 0.445–0.604, *p* < 0.001) (Supplemental Figure 2). For the full sample of 693 patients, the AUROC was 0.908 (*95% CI* 0.881–0.931), which was higher than the CURB-65 score (AUC = 0.777, *95% CI* 0.740–0.811, *p* < 0.001) and the PSI risk class (AUC = 0.560, *95% CI* 0.518–0.602, *p* < 0.001) (Table 2 and Fig. 3). Table 3 shows the sensitivity, specificity and actual mortality associated with the FluA-p score (in the full sample of 693 patients). In accordance with the cut-score approach described earlier, patients were divided into high-risk and low-risk groups based on a cut-off value of 7. The Kaplan-Meier survival curves showed that 30-day mortality was significantly higher in patients with high-risk than for patients at low-risk (52.9% vs 2.1%, log rank test, *p* < 0.001) (Fig. 4).

**Table 2:** AUC for mortality predictions in FluA-p patients

Variable	AUC	SE	*95% CI*	*Z* statistic	*p* value
FluA-p score	0.908	0.016	0.881–0.931	–	Reference
PSI risk class	0.560	0.035	0.518–0.602	10.875	< 0.001
CURB-65 score	0.777	0.020	0.740–0.811	6.041	< 0.001

*AUC* area under the curve, *SE* standard error, *CI* confidence interval

**Table 3:** FluA-p score and actual mortality

**Score**	**Actual 30-day** **Mortality (*n, %*)**	**Sensitivity**	***95% CI***	**Specificity**	***95% CI***	**+LR**	**-LR**
-2 ~ 1	0/120 (0.0)	100.00	97.3–100.0	0.00	0.0–0.8	1.00	
2	8/16 (50.0)	100.00	97.3–100.0	25.05	21.2–29.2	1.33	0.00
3 ~ 6	0/237 (0.0)	94.12	88.7–97.4	26.72	22.8–30.9	1.28	0.22
**7**	20/82 (24.4)	94.12	88.7–97.4	76.20	72.1–79.9	3.95	0.077
8	8/32 (25.0)	79.41	71.6–85.9	89.14	86.0–91.8	7.32	0.23
9	40/56 (71.4)	73.53	65.3–80.7	94.15	91.7–96.1	12.58	0.28
10	8/8 (100.0)	44.12	35.6–52.9	97.49	95.7–98.7	17.61	0.57
11	52/64 (81.3)	38.24	30.0–47.0	97.49	95.7–98.7	15.26	0.63
12	NA	0.00	0.0–2.7	100.00	99.2–100.0		1.00

*+LR* positive likelihood ratio, *−LR* negative likelihood ratio

## Discussion

Our study not only assessed several risk factors, but also developed a simple and reliable prediction tool for predicting mortality in patients with FluA-p. Our method showed greater predictive validity than did the common pneumonia severity scores of PSI and CURB-65.

PSI and CURB-65 scores are recommended by the Infectious Diseases Society of America/American Thoracic Society (IDSA/ATS) and the British Thoracic Society (BTS) for the assessment of disease severity of CAP [20, 21]. Numerous studies have found that PSI and CURB-65 scores accurately predict the 30-day mortality rates of CAP and are applicable for use in many clinical settings [22–24]. Recently, however, some studies suggested that they were insufficient for predicting mortality in settings involving influenza pneumonia [13–16]. Our results likewise suggested that PSI and CURB-65 scores underestimated the mortality of FluA-p patients. More than half of the deceased patients were classified as low death risk (CURB-65 score 0–2 and PSI risk class I~III). Both CURB-65 and PSI were heavily weighted by age and comorbidities. But many Flu-p patients were young and previously healthy individuals. In our study cohort, 60% of patients were younger than 65 years of age. During the H1N1 influenza A pandemic in 2009, a large proportion of severe cases were young patients who experienced acute respiratory failure [25, 26]. Another issue to consider is that the current severity tool that relies on PSI and CURB-65 scores was possibly derived from patients diagnosed primarily with bacterial and atypical bacterial pneumonia rather than influenza pneumonia [20, 27]. In fact, Guo et al. reported that CURB-65 scores were not powerful predictors of mortality in the context of viral pneumonia [28].

Several studies have reported lymphocytopenia in severe influenza [27, 29, 30]. Shi et al. suggested that lymphocytopenia was an early and reliable predictor of mortality in patients diagnosed with influenza A(H1N1)pdm09 pneumonia [27]. Although the mechanisms of lymphocytopenia in severe influenza are not well elucidated, it is believed that the reduction of T lymphocytes (including CD8 + T effector and central memory cells, CD4 + T, and/or NK cells), rather than B lymphocytes, in the peripheral blood might be the causes of lymphocytopenia [31, 32]. Lymphocytopenia also plays a role in suppressive cellular immunity and the delayed clearance of viruses [33].

Smoking history was another pedictor of FluA-p mortality in our study, which is a finding commensurate with some previous reports [34–36]. Wong and colleagues, for example, found that influenza-related mortality for all-causes and for cardiovascular and respiratory diseases was greater in current and ex-smokers than in never smokers [34]. A case-control study by Hennessy et al. found that smoking (*OR* 3.03, *95% CI* 1.01–9.23) was a significant risk factor for death in patients with A(H1N1) pdm09 [35]. Although the precise nature of the association between smoking and influenza-related mortality has yet to be determined, some potential mechanisms suggest the possibility of biological associations. Smoking could disrupt the normal defenses of the respiratory tract by causing peribronchiolar inflammation, slowing mucociliary clearance, and/or damaging respiratory epithelial cells [37]. Animal studies using mouse models have shown that smoking induces inflammatory mediators and suppresses innate immunity against influenza infection [38]. Smoking could increase viral replication by directly suppressing epithelial antiviral pathways, facilitating cytokine release in mucosal innate immunity and increasing deoxyribonucleic acid (DNA) methylation for viral infection [39].

BUN, pO_2_/FiO_2_, and arterial PH were parameters in calculating PSI and/or CURB-65 scores. Our study showed that these parameters were valuable predictors of mortality in FluA-p patients. Early administration of NAI therapy is associated with better outcomes in severe influenza [40, 41]. Old age, obesity, pregnancy and chronic medical conditions, such as COPD, diabetes mellitus, and chronic kidney disease, have been associated with poorer outcomes in patients with influenza [35, 42, 43]. However, in our study only cardiovascular disease was identified as a risk factor for mortality in FluA-p patients. Other studies have shown that coinfections can worsen illness severity and increase mortality in severe influenza [44, 45]. In our univariate analyses, coinfections were associated with increased mortality for FluA-p patients, but coinfections were not significant predictors in the multivariate analysis.

FluA-p score is a very simple severity assessment tool containing only seven parameters and it serves as a reliable prediction rule. ROC showed better predictive validity compared to PSI risk class and the CURB-65 score. Although the specificity of score 2 is not good (only 25%), judging from the performance of score − 2 ~ 1 and score 3 ~ 6, we believe it is mainly because patients with score 2 were scarce (only 16 cases) in our study. Larger subgroup sample sizes would allow for stronger inferences Using a cutoff value of 7, the new FluA-p score could stratify patients into two groups with significantly different death risks. We believe this novel assessment tool is suitable for use in clinical settings with FluA-p patients. In addition, the parameters include indicators widely used in clinics, even in small and perhaps less equipped hospitals. Consequently, we think the assessment tool has a great practical value.

Some limitations of our study should be noted. First, despite our respectable sample size and comprehensive statistical approach, the retrospective research design meant some unavoidable selection bias. For example, the nucleic acid tests were performed based on the subjective judgement of the attending physicians. It was possible that more severe (or milder) patients were inclined to be tested; thus, not all respiratory cases were eligible for swabbing and there was likely some type of selection. Second, due to the retrospective study design, we were unable to retrieve and evaluate vaccination data, and the incomplete data might have lowered the accuracy of our results. Finally, some studies have suggested that the clinical characteristics and prognosis of immunocompromised patients with influenza is not the same as that for immunocompetent hosts [46, 47]. Thus, it is important to further assess our influenza prediction model in immunocompromised patients.

## Conclusions

We developed a simple and reliable prediction rule for 30-day mortality in patients hospitalised with FluA-p. The prediction rule could help clinicians to more accurately assess influenza disease severity. Our recommendation is that clinicians should pay particular attention to patients with FluA-p scores ≥7, as such individuals have an increased risk for death.

## Supplementary information

**Additional file 1.** Supplementary material 1: Details of participating centers. Supplementary material 2 Definition of underlying diseases. Supplementary material 3 Definition of microbiological criteria of coinfected with other pathogens. Supplementary material 4 coinfections with other community-acquired pathogens. Supplemental material 5 Empirical antibiotics therapy regimes. Supplementary material 6 Comparison of baseline clinical characteristics and outcomes between the derivation and validation cohort. Supplementary material 7 Predicted and actual mortality rates in FluA-p patients stratified by two common severity scores. Supplementary material 8 AUC for mortality prediction in FluA-p patients from derivation cohort. Supplementary material 9 AUC for mortality prediction in FluA-p patients from validation cohort. **Figure S1.** ROCs for mortality prediction of three severity scores in FluA-p patients from derivation cohort. **Figure S2.** ROCs for mortality prediction of three severity scores in FluA-p patients from validation cohort.

## Data Availability

All data generated or analysed during this study are included in this published article [and its supplementary information materials].

## Abbreviations

Flu-p: Influenza-related pneumonia
FluA-p: Influenza A-related pneumonia
PSI: Pneumonia severity index
CURB-65: Confusion, urea, respiratory rate, blood pressure, age ≥ 65 years
NAI: Neuraminidase inhibitor
OR: Odds ratio
IC: Interval confidence
ROC: Receiver operating characteristic
AUROC: Area under the ROC curve
COPD: Chronic obstructive pulmonary disease
SBP: Systolic blood pressure
Hb: Hemoglobin
ALB: Albumin
BUN: Blood urea nitrogen
PH: Hydrogen ion index
pO_2_/FiO_2_: Arterial pressure of oxygen/fraction of inspiration oxygen
ICU: Intensive care unit
IDSA/ATS: Infectious Diseases Society of America/American Thoracic Society
BTS: British Thoracic Society
DNA: deoxyribonucleic acid
