# Supplementary material for: FluA-p score: a novel prediction rule for mortality in influenza A-related pneumonia patients
Source: Respir Res. 2020 May 8;21:109. doi: 10.1186/s12931-020-01379-z (PMC7206684; doi:10.1186/s12931-020-01379-z)
Supplement: Supplementary file 1 — Additional file 1. Supplementary material 1: Details of participating centers. Supplementary material 2 Definition of underlying diseases. Supplementary material 3 Definition of microbiological criteria of coinfected with other pathogens. Supplementary material 4 coinfections with other community-acquired pathogens. Supplemental material 5 Empirical antibiotics therapy regimes. Supplementary material 6 Comparison of baseline clinical characteristics and outcomes between the derivation and validation cohort. Supplementary material 7 Predicted and actual mortality rates in FluA-p patients stratified by two common severity scores. Supplementary material 8 AUC for mortality prediction in FluA-p patients from derivation cohort. Supplementary material 9 AUC for mortality prediction in FluA-p patients from validation cohort. Figure S1. ROCs for mortality prediction of three severity scores in FluA-p patients from derivation cohort. Figure S2. ROCs for mortality prediction of three severity scores in FluA-p patients from validation cohort. [file 12931_2020_1379_MOESM1_ESM.doc]

**Supplementary material 1: Details of participating centers**

| **Name of the hospital** | **Province, city** | **Teaching Hospital** | **Beds** | **Staffs of Clinical Microbioloy Lab** |
| --- | --- | --- | --- | --- |
| Beijing Jishuitan Hospital, | Beijing | Yes | 1500 | 10 |
| Beijing Chao-Yang Hospital | Beijing | Yes | 1400 | 11 |
| **the 2nd People’s Hospital of Yunnan Province** | **Kunming,**  **Yan’an** | Yes | 1302 | 4 |
| **Qingdao Municipal Hospital** | ShanDong,  Qingdao | Yes | 1200 | 4 |
| Beijing Huimin Hospital | Beijing | Yes | 1000 | 2 |

**Supplementary material 2 Definition of underlying diseases**

1. Smoking was defined as cigarette smokers of 10 cigarettes/d during at least the previous year；
2. Hypertension was defined as systolic blood pressure≥140mmHg and /or diastolic blood pressure ≥ 90 mmHg in resting status;
3. Chronic pulmonary disease was defined as: persistent airflow limitation, FEV_1_ / FVC < 70% post bronchodilator;
4. Asthma was defined by the history of respiratory symptoms such as wheeze, cough that varied over time and intensity, together with variable respiratory airway limitation;
5. Cardiovascular disease included coronary heart disease and chronic congestive heart failure;
6. Coronary heart disease included angina pectoris, myocardial infarction, ischemic cardiomyopathy;
7. Chronic congestive heart failure was defined as cardiomegaly and ejection fraction ≤ 40%;
8. Cerebrovascular diseases included transient ischemic attack, cerebral hemorrhage, subarachnoid hemorrhage, cerebral infarction;
9. Diabetes mellitus: included diabetes mellitus type 1 and diabetes mellitus type 2, not included impaired glucose tolerance and impaired fasting glycaemia;
10. Chronic kidney disease included diabetic nephropathy, hypertensive renal damage, chronic glomerulonephritis, chronic pyelonephritis, lupus nephritis, IgA nephropathy, nephrotic syndrome, hereditary kidney disease;
11. Obesity was defined as body mass index (BMI) ≥ 30 kg/m^2^;
12. Immunosuppressive therapy: was defined as systmetic glucocorticosteroid (such as prednisone ≥ 10mg/d for more than 3 weeks in the last month); cyclosporine or azathioprine use within 3 months, and methotrexate use ≥ 12.5 mg/week within 3 months; biological modifiers such as etanercept and infiximab within 3 weeks.
13. Immunocompromised status included HIV(+), chemotherapy/radiotherapy within 6 months, immunosuppressive therapy, organ/bone marrow transplantation, splenectomy, hematological neoplasms.
14. Mental confusion was defined as a mental test score of 8 or less or disorientation in person, place or time.

**Supplementary material 3 Definition of microbiological criteria of coinfected with other pathogens**

1. Positive urinary antigen for *Legionella pneumophila*;
2. Positive urinary antigen for *Streptococcus pneumoniae*;
3. Positive bacterial culture from blood or plural fluid except for coagulase negative *Staphylococcus spp*.;
4. Paired sera with a fourfold or more increase in the titers of antibodies to *Mycoplasma pneumoniae* (MP), *Chlamydia pneumonia*, *L pneumophila or* respiratory viruses (Parainfluenza, Adenovirus, Respiratory syncytial virus)*.* Or Serum IgM antibody (MIF) ≥ 1:16 for *Chlamydia pneumonia*;
5. Detection of respiratory virus in sputum/bronchoalveolar lavage (BALF)/throat swabs by Realtime-PCR according to manufacturer’s instructions, including respiratory syncytial virus (RSV) types A and B, parainfluenza virus (PIV) types 1, 2, 3 and 4, rhinovirus (HRV), enterovirus (EV), coronavirus (hCoV) types 229E, NL63, OC43 and HKU1, parapneumovirus (hMPV), and adenovirus (AdV), bocavirus;
6. Bacteria isolated form purulent sputum (defined as an adequate quality sputum sample with > 25 leukocytes and < 10 epithelial cells per × 100 magnification field) with compatible findings of Gram staining;
7. Detection of *Mycoplasma pneumoniae* (MP), *Chlamydia pneumonia* or *L pneumophila* in sputum/BALF/throat swabs by Realtime-PCR;
8. serum IgM antibody positive for *Mycoplasma pneumoniae* (MP), or Serum IgG antibody (MIF) ≥ 1:512 for *Chlamydia pneumonia;*
9. Invasive pulmonary aspergillosis were diagnosed in accordance with the revised definitions of invasive fungal diseases from the European Organization for Research and Treatment of Cancer and the Mycoses Study Group Education and Research Consortium [1].

References

1.Donnelly JP, Chen SC, Kauffman CA, et al. Revision and Update of the Consensus Definitions of Invasive Fungal Disease From the European Organization for Research and Treatment of Cancer and the Mycoses Study Group Education and Research Consortium. Clin Infect Dis. 2019, pii: ciz1008.

**Supplementary material 4 coinfections with other community-acquired pathogens**

| Variable | Total  (*n* = 693) |
| --- | --- |
| Coinfection (*n*,%) | 265 (38.2) |
| Pathogens (*n*,%) |  |
| *Streptococcus pneumoniae* | 88 (33.2) |
| *Klebsiella pneumoniae* | 81 (30.6) |
| *Staphylococcus aureus* | 54 (20.4) |
| *Haemophilus influenzae* | 17 (6.4) |
| *Pseudomonas aeruginosa* | 12 (4.5) |
| *Klebsiella acidogens* | 7 (2.6) |
| *Acinetobacter* | 5 (1.9) |
| *Proteus spp.* | 3 (1.1) |
| *Stenotrophomonas maltophilia* | 3 (1.1) |
| *Escherichia coli* | 2 (0.8) |
| *Aspergillus spp.* | 2 (0.8) |
| *Citrobacter spp.* | 1 (0.3) |

10 patients were coinfected with ≥ 2 pathogens

**Supplemental material 5 Empirical antibiotics therapy regimes**

| Empirical antibiotics therapy regimes | Patients (*n*, %) |
| --- | --- |
| Macrolides | 23 (3.3) |
| Fluoquinolones | 257 (37.1) |
| β-lactams | 211 (30.4) |
| β-lactams+ fluoquinolones | 104 (15.0) |
| β-lactams+ macrolides | 98 (14.1) |

**Supplementary material 6 Comparison of baseline clinical characteristics and outcomes between the derivation and validation cohort**

| **Variable** | **Derivation Cohort**  **(*n* = 494)** | **Validation Cohort**  **(*n* = 199)** | ***p* value** |
| --- | --- | --- | --- |
| Age (years, median, IQR) | 59.0 (36.0-72.3) | 62.0 (34.0-76.0) | 0.208 |
| Male (*n*, %) | 327 (66.2) | 134 (67.3) | 0.773 |
| Chronic medical conditions (*n*, %) |  |  |  |
| Cardiovascular disease | 97 (19.6) | 45 (22.6) | 0.380 |
| Cerebrovacular disease | 49 (9.9) | 23 (11.6) | 0.522 |
| Diabetes mellitus | 73 (14.8) | 19 (9.5) | 0.066 |
| COPD | 24 (4.9) | 17 (8.5) | 0.063 |
| Asthma | 21 (4.3) | 6 (3.0) | 0.447 |
| Chronic kidney disease | 12 (2.4) | 4 (2.0) | 0.958 |
| Malignant solid tumor | 8 (1.6) | 10 (5.0) | 0.011 |
| Obesity (n,%) | 37 (7.5) | 11 (5.5) | 0.357 |
| Pregnancy (n,%) | 5 (1.0) | 3 (1.5) | 0.873 |
| Smoking history (n,%) | 176 (35.6) | 67 (33.7) | 0.625 |
| Baseline clinical and radiological features |  |  |  |
| Altered mental status (*n*, %) | 26 (5.3) | 6 (3.0) | 0.202 |
| Respiratory rates ≥ 30 beats/min (*n*, %) | 82 (16.6) | 39 (19.6) | 0.432 |
| SBP < 90 mmHg (*n*, %) | 6 (1.2) | 2 (1.0) | 1.000 |
| Leukocytes (×10^9^/L, mean ± SD) | 6.8±3.4 | 7.1±4.0 | 0.335 |
| Lymphocytes (×10^9^/L, mean ± SD) | 1.0±0.7, *n* = 481 | 1.0±0.6, *n* = 196 | 0.158 |
| HB (g/L, mean ± SD) | 131.6±22.2 | 132.4±21.7 | 0.632 |
| ALB (g/L, mean ± SD) | 29.1±5.8, *n* = 446 | 29.0±6.3, *n* = 183 | 0.905 |
| BUN (mmol/L, mean ± SD) | 6.0±4.1, *n* = 489 | 5.8±3.8, *n* = 196 | 0.687 |
| pO_2_/FiO_2_ (mmHg, mean ± SD) | 316.5±132.7, *n* = 457 | 316.6±129.7, *n* =182 | 0.988 |
| Coinfections (*n*, %) | 179 (36.2) | 86 (43.2) | 0.087 |
| Early NAI therapy (*n*, %) | 164 (33.2) | 68 (34.2) | 0.806 |
| Systemic corticosteroid use (*n*, %) | 99 (20.0) | 33 (16.6) | 0.294 |
| Noninvasive ventilation (*n*, %) | 106 (21.5) | 53 (26.6) | 0.277 |
| Invasive ventilation (*n*, %) | 104 (21.1) | 54 (27.1) | 0.084 |
| Admittance to ICU (*n*, %) | 125 (25.3) | 51 (25.6) | 0.929 |
| 30-day mortality (*n*, %) | 91 (18.4) | 45 (22.6) | 0.209 |

IQR: interquartile range; SD: standard deviation; COPD: chronic obstructive pulmonary disease; SBP: systolic blood pressure; Hb: hemoglobin; ALB: albumin; BUN: blood urea nitrogen; BG: blood glucose; pO_2_/FiO_2_: arterial pressure of oxygen/fraction of inspiration oxygen; NAI: neuraminidase inhibitor; ICU: intensive care unit;.

## **Supplementary material 7** Predicted and actual mortality rates in FluA-p patients stratified by two common severity scores

| **Risk stratification** | **Total patients (n)** | **Deceased patients (n)** | **Actual mortality (%)** | **Predicted mortality (%)** |
| --- | --- | --- | --- | --- |
| PSI risk class |  |  |  |  |
| I | 176 | 52 | 29.5 | 0.1 |
| II | 183 | 12 | 6.6 | 0.6 |
| III | 136 | 8 | 5.9 | 0.9 |
| IV | 88 | 64 | 72.7 | 9.3 |
| V | 16 | 0 | 0 | 27.0 |
| CURB-65 score |  |  |  |  |
| 0 | 253 | 0 | 0 | 0.7 |
| 1 | 283 | 91 | 32.2 | 2.1 |
| 2 | 118 | 17 | 14.4 | 9.2 |
| 3 | 31 | 28 | 90.3 | 14.5 |
| 4 | 0 | 0 | - | 40.0 |
| 5 | 0 | 0 | - | 14.0 |

**Supplementary material 8 AUC for mortality prediction in FluA-p patients from derivation cohort**

|  | **AUC** | **SE** | ***95% CI*** | ***Z* statistic** | ***p* value** |
| --- | --- | --- | --- | --- | --- |
| FluA-p score | 0.934 | 0.016 | 0.906 - 0.957 | —— | Reference |
| PSI risk class | 0.577 | 0.043 | 0.527 - 0.625 | 8.920 | < 0.001 |
| CURB-65 score | 0.813 | 0.022 | 0.772 - 0.850 | 5.235 | < 0.001 |

AUC: area under the curve; SE: standard error; CI:confidence interval.

**Supplementary material 9 AUC for mortality prediction in FluA-p patients from validation cohort**

|  | **AUC** | **SE** | ***95% CI*** | ***Z* statistic** | ***p* value** |
| --- | --- | --- | --- | --- | --- |
| FluA-p score | 0.846 | 0.038 | 0.781 - 0.897 | —— | Reference |
| PSI risk class | 0.525 | 0.058 | 0.445 - 0.604 | 5.981 | < 0.001 |
| CURB-65 score | 0.681 | 0.040 | 0.604 - 0.752 | 3.533 | < 0.001 |

AUC: area under the curve; SE: standard error; CI:confidence interval.


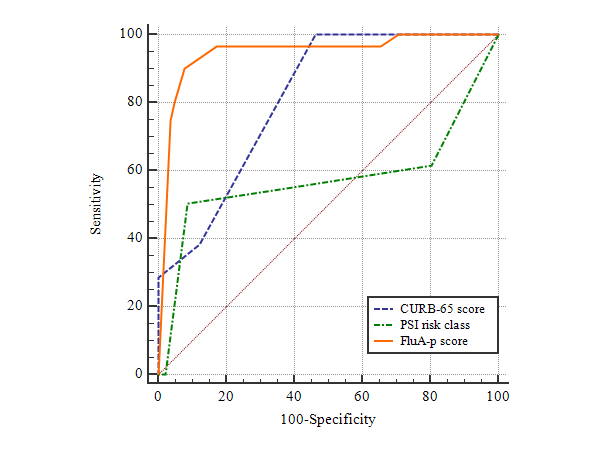


**Supplementary figure 1 ROCs for mortality prediction of three severity scores in FluA-p patients from derivation cohort**


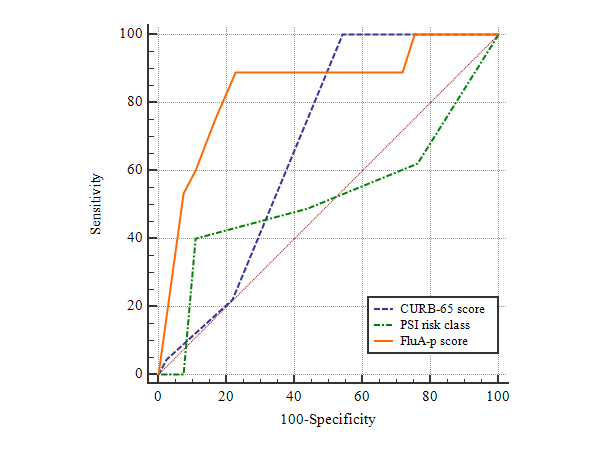


**Supplementary figure 2 ROCs for mortality prediction of three severity scores in FluA-p patients from validation cohort**
